# Transperineal cryotherapy for unresectable muscle invasive bladder cancer: preliminary experience with 7 male patients

**DOI:** 10.1186/s12894-017-0270-y

**Published:** 2017-09-09

**Authors:** Qing Zhang, Shiwei Zhang, Shun Zhang, Wei Wang, Xiaozhi Zhao, Yongming Deng, Huibo Lian, Hongqian Guo

**Affiliations:** Department of Urology, Drum Tower Hospital, Medical School of Nanjing University, Institute of Urology, Nanjing University, 321 Zhongshan Road, Nanjing, 210008 Jiangsu People’s Republic of China

**Keywords:** Cryotherapy, Invasive bladder cancer, Progression free survival, Transperineal cryoablation, Unresectable

## Abstract

**Background:**

Radical cystectomy (RC) with pelvic lymph node dissection (PLND) and urinary diversion (UD) is considered the standard treatment for muscle invasive bladder cancer (MIBC). In a part of patients, RC procedure is aborted due to unresectable disease, other followed treatment like systemic chemotherapy, radiotherapy or cryotherapy may be a better option. The aim of present study was to report the preliminary results of transperineal cryotherapy for unresectable muscle invasive bladder cancer.

**Methods:**

From January 2011 to August 2013, 7 male patients with pT4b unresectable bladder cancer underwent bilateral ureterocutaneostomy. Two performed a pelvic lymph node dissection (PLND). Then primary transperineal cryosurgery for preserved bladder at the guidance of transrectal ultrasound (TRUS) was performed. All patients underwent a dual freeze-thaw cycle using third-generation cryotechnology with ultrathin 17-gauge cryoneedles. Computer tomography (CT) and/or magnetic resonance image (MRI)were performed at 3 month intervals after cryosurgery to determine whether progression or recurrence occurred.

**Results:**

All cryosurgery was performed successfully, mean operation time was 76.43 ± 25.12 min (range 50-120 min), mean blood loss was 19.29 ± 15.92 ml (range 5-50 ml). Mean hospital stay was 3.86 ± 1.68 day (range 2-7 days). No operative related deaths occurred. Four patients dead due to the metastasis disease at the follow up time of 8, 15, 18 and 37 months, respectively. Six patients received postoperative therapy, of whom 5 patients were treated with combined chemoradiation, and the other one received chemotherapy alone. The progression free survival (PFS) of the 7 patients was 22.00 ± 14.61 months (range 3-40 months). The one, two and three year overall survival (OS) was 85.7%, 57.1% and 42.9%, respectively.

**Conclusion:**

Our results suggest that cryosurgery combination with chemoradiotherapy provide a safe and effective alternative method for unresectable pT4b bladder cancer. Longer follow-up is necessary to determine the sustained efficacy.

## Background

Bladder cancer (BC) is a common human malignancy and is the second most common genitourinary malignancy [1]. About 20-40% of patients with BC present or develop a muscle invasive bladder cancer (MIBC). Currently, radical cystectomy (RC) with pelvic lymph node dissection (PLND) and urinary diversion (UD), although associated with significant morbidity, is considered the standard treatment for MIBC [2]. In a part of patients, RC procedure is aborted due to unresectable disease, either the presence of gross palpable nodes (pN2-3) or tumor fixation to the pelvis or rectum (pT4b). In these patients, RC will be aborted intraoperatively, other followed treatment like systemic chemotherapy, radiotherapy or cryotherapy may be a better option.

Cryosurgery, which can induce tissue necrosis by ice ball formation, has been used as an alternative therapeutic approach in bladder cancer for half a century. The effects of cryoablation on the bladder were investigated in dogs by McDonald et al. in 1950 and typical necrotic lesions were produced [3]. Several reports recently described the utility of cryosurgery in the treatment of bladder cancer through percutaneous or transurethral route and concluded that this precudure could be used as a novel therapeutic approach to the treatment of benign and malignant bladder tumors, especially unresectable tumors and metastatic bladder cancer [4, 5].

Since 2011, we have performed transperineal cryotherapy in patients who had aborted RC with unresectable disease secondary to fixation to the pelvis and/or rectum and followed ureterocutaneostomy. The aim of the current study was to analyze the preliminary results and to investigate the safety and efficacy of transperineal cryotherapy in our 7 male patients.

## Methods

### Patients

**Fig. 1:**
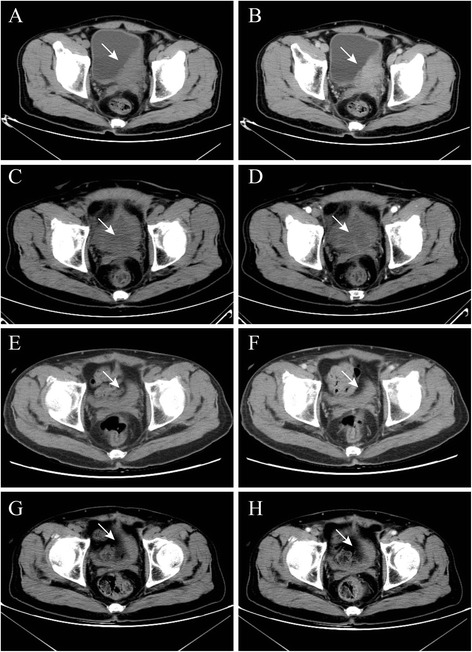
CT images of a patient with cancer in the left wall of the bladder (patient number 5) Non-contrast (**a**) and contrast-enhanced arterial phase images (**b**) of CT scans indicated that the tumor (white arrow) located on left wall of bladder, the longest diameter of tumor was 8.1 cm before cryosurgery. One months after cryosurgery, non-contrast (**c**) and contrast-enhanced arterial phase images (**d**) of CT show the structure of the bladder wall remained intact and tumor (white arrow) inside and outside the bladder had no obviously enchancement. One year after cryosurgery, non-contrast (**e**) and contrast-enhanced arterial phase CT images (**f**) show the bladder (white arrow) contraction and no urinary filling. There was no obviously enchancement in the preserved bladder. Three year after cryosurgery, non-contrast (**g**) and contrast-enhanced arterial phase images (**h**) of CT show the bladder (white arrow) shrink further and no obviously enchancement in the shrinked bladder. No progression and local recurrence happened in this patient at the follow up time of 40 months

Between January 2011 to August 2013, 7 male patients with unresectable bladder cancer underwent transperineal cryotherapy in our institution were enrolled in the study. All patients had an aborted RC due to unresectable disease secondary to fixation to the pelvis and/or rectum (pT4b) then the followed ureterocutaneostomy was performed. Preoperative work-ups were performed, including serum blood tests, chest X-ray, abdomen/pelvis computed tomography scans (CT) (Fig. 1a and b), and bone scans if clinically indicated. Moreover, each patient underwent a staging cystoscopy with transurethral resection of bladder tumor (TURBT). Preoperative CT and/or MRI scans were negative for pelvic adenopathy, metastasis, or evidence of clinically unresectable disease (Fig. 1a and b). The pre-anesthetic risk was quantified using the American Society of Anesthesiologist’s physical status classification score. No neoadjuvant chemotherapy was applied. Then cryoablation was performed on the preserved bladder at the second time and the followed adjuvant chemoradiotherapy or chemotherapy alone was performed (Gemcitabine and cisplatin regimens chemotherapy were added to patients 3 months later after cryoablation, followed local radiotherapy for bladder cancer). Each patient provided written informed consent, and the study was approved by Ethics Committee of Nanjing Drum Tower Hospital. Patient demographics were summarized in Table 1.

**Table 1:** Data for 7 male patients (age range from 47 to 73 years) who underwent transperineal cryotherapy for high grade MIBC

Patients	TLD(cm)	OP(min)	PFS(month)	Location	Initial symptom	Complications Post-cryosurgery	Additional Treatment
1	7.5	55	11	Left wall	Hematuria	None	Chemoradiotherapy
2	8.3	70	25	Left wall	Urinary irritation	Urinary irritation	Chemoradiotherapy
3	6.5	50	35	Left wall	Hematuria, Urinary irritation, Abdominal pain	Abdominal pain	Chemotherapy
4	11.4	120	3	Right wall	Hematuria	None	None
5	8.1	100	40	Left wall	Hematuria, Abdominal pain	None	Chemoradiotherapy
6	5.8	75	8	Back wall	Hematuria	None	Chemoradiotherapy
7	7.5	65	32	Right wall	Hematuria, Urinary irritation	None	Chemoradiotherapy

Note: *TLD* tumor longest diameter, *PFS* progression free survival, *OP* operation time

### Ureterocutaneostomy and PLND

RC with appropriate LND and UD was the preferred treatment for patients with MIBC. However, RC could not be achieved due to the diseases of the fixation to the pelvis and/or rectum (pT4b) or the presence of gross palpable nodes (pN2-3) according intraoperative findings. All the patients were pT4b stage and urinary diversion was performed with bilateral ureterocutaneostomy. Two of the 7 patients had a PLND, and 5 did not at the discretion of the treating surgeon in our study. Ureterocutaneous anastomosis was performed according to the technique originally described by Glenn [6].

### Cryosurgery procedure

**Fig. 2:**
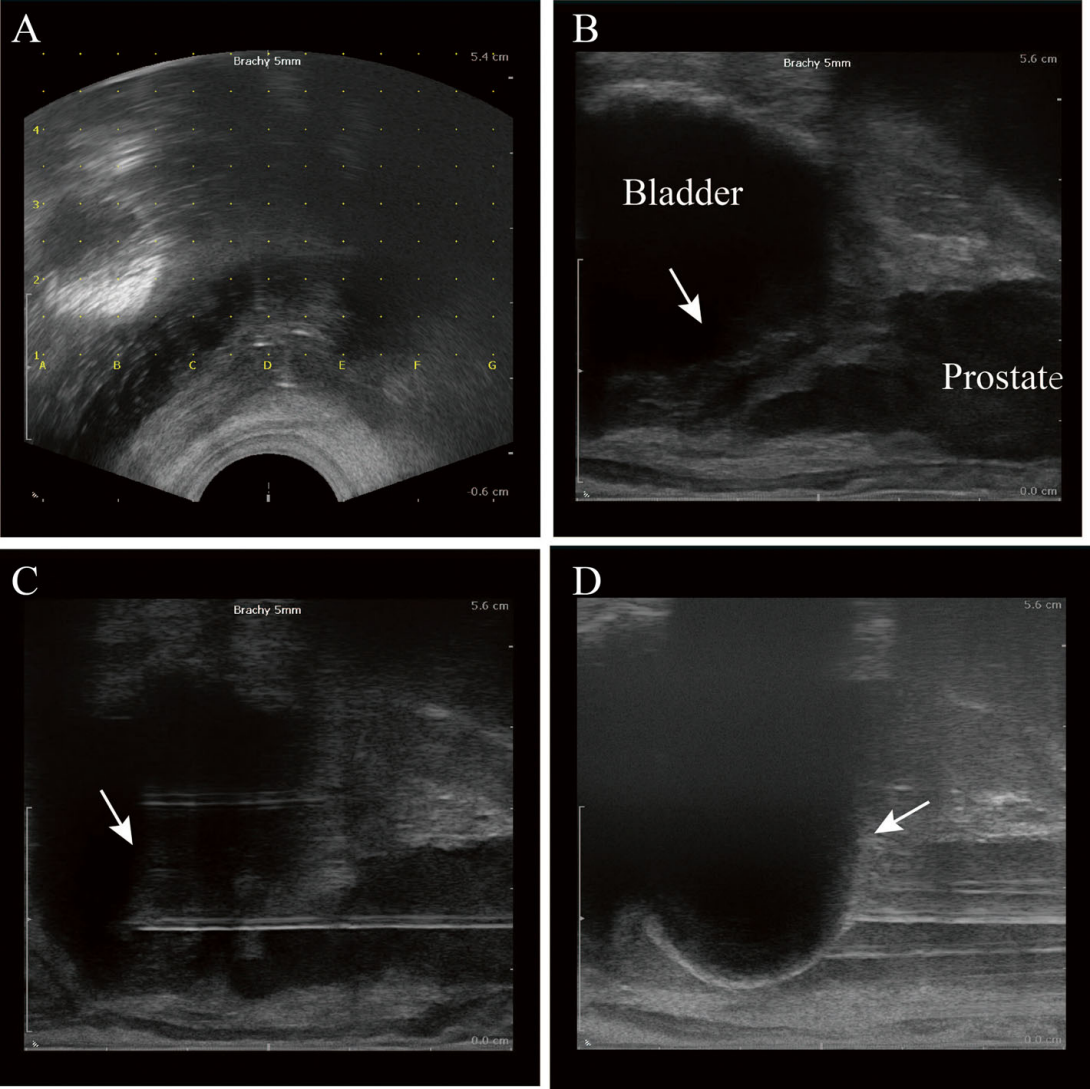
Images of transperineal cryotherapy procedure for unresectable muscle invasive bladder cancer under the real-time guidance of TRUS (patient number 5). The template was placed in stepper, approximate to perineum, and the grid was overlaid on ultrasound screen (**a**). Bladder tumor (white arrow) and other nearby tissues and organs including prostate and rectum were identified by TRUS (**b**). Under TRUS guidance, two 1.47-mm 17G IceSeed cryoneedles were placed transperineally into the bladder tumor (white arrow) (**c**). Two single-point temperature monitoring probes were placed at tumor around and rectum, respectively. Freezing with argon gas to < − 40 °C was initiated and monitored using the temperature-monitoring probes, and TRUS guidance, which revealed an acoustic shadow as the ice-ball formed (white arrow) (**d**)

All cryosurgery procedures were performed using the Cryo-Hit System (Galil Medical Ltd., Israel) by a single urologist. All patients were placed in a modified lithotomy position under spinal anesthesia. The scrotum was elevated off the perineum after a 18F Foley catheter was inserted into bladder. The template was placed in stepper, approximate to perineum, and the grid was overlaid on ultrasound screen (Fig. 2a). The template and ultrasound grid were verified alignment with first needle. All needles were tested before insertion. Under transrectal ultrasound (TRUS) guidance, 1.47-mm 17G IceSeed cryoneedles were placed transperineally into the bladder tumor (Fig. 2b and c). Two single-point temperature monitoring probes were placed at tumor around and rectum, respectively. The number and position of cryoneedles varied with bladder cancer size and location. Freezing with argon gas to < − 40 °C was initiated and monitored using the temperature-monitoring probes, and TRUS guidance, which revealed an acoustic shadow as the ice-ball formed (Fig. 2d). The tumor around and rectum was maintained at temperatures greater than 0 °C, and freezing was stopped when the iceball reached the anterior rectal wall was at least 20 °C. After maintaining −40 °C or the lowest temperature below −25°Cfor 10 min, a passive thaw was initiated until the temperature reached a plateau. At this point, active thawing with helium was started. Two cycles of rapid freeze-thaw were carried out, ensuring the temperature in the bladder cancer and just outside it was below a therapeutic value of −40 °C. At the end of the procedure, the needles and probes were removed and pressure applied to the perineal area for 10 min to reduce bruising.

### Follow up evaluation and statistical analysis

Radiographic local tumor control was assessed using image-guided tumor ablation criteria [7]. Follow-up CT and MRI were performed at 3-month intervals after cryotherapy procedure. Two diagnostic radiologists reviewed CT and MRI scans for every case to determine whether progression or recurrence had occurred. Progression free survival (PFS) and overall survival (OS) were calculated from the date of cryosurgery. Data are presented as the mean ± standard deviation (SD). Analyses were performed with Statistical Package for Social Sciences, version 17 (SPSS, Chicago, IL).

## Results

Totally 7 male patients were included in this study. The clinical characteristics of the 7 patients are summarized in Table 1. The mean age of patients was 60.14 ± 9.04 years (range from 47 to 73 years) with a mean follow-up of patients alive of 28.43 ± 14.25 months (range from 8 to 43 months). Five patients were found to have hydronephrosis, either unilateral or bilateral. Histology showed all these patients were high grade urothelial carcinoma according to TURBT and the followed biopsy. Tumors were located in the right, left or back wall of the bladder in two, four, and one cases, respectively. The mean tumor longest diameter was 7.87 ± 1.79 cm (range from 5.8 to 11.4 cm). All 7 patients planned to cystectomy, but underwent aborted cystectomy due to unresectable disease secondary to fixation to the pelvis and/or rectum (pT4b stage) according to the intraoperative findings and the followed ureterocutaneostomy was performed. Mean time from ureterocutaneostomy to cryosurgery was 6.00 ± 1.63 months (ranging from 4 to 9 months). Two of the 7 patients had a PLND, and the other 5 had no PLND at the discretion of the treating surgeon. No positive pelvic lymph nodes were found in followed histopathological examination of the two patients.

All the cryosurgery procedures were performed successfully and all patients well tolerated with the transperineal cryosurgery, and with minimal requirements for pain medication and without the need for blood transfusions. No operative-related deaths occurred in these patients. The mean operative time of transperineal cryosurgery was 76.43 ± 25.12 min (range from 50 to 120 min). The mean bleed loss was 19.29 ± 15.92 ml (range from 5 to 50 ml). The mean length of hospital stay for the patients after cryosurgery was 3.86 ± 1.68 days (range from 2 to 7 days). In the process of follow-up, all patients have abandoned freezing treatment again, and two patients’ dead due to pulmonary metastasis disease at the follow up time of 8 and 37 months, the other two patients’ dead due to extensive abdominal and pelvic metastasis at 15 and 18 months after cryosurgery respectively. The other three patients were alive at 43, 40 and 38 months after cryosurgery and no progression of disease at the follow up examination of CT (Fig. 1c–h). There were minor complications but no major complications of the treatment. These new treatment-related symptoms are not considered as complications and no other minor or severe complications were noticed. All complications had disappeared completely after 2 weeks. Six of 7 patients received postoperative therapy, of whom 5 patients were treated with combined identical chemoradiation, while the other one received chemotherapy alone. The PFS of the 7 patients was 22.00 ± 14.61 months (range 3-40 months). The one, two and three-year OS was 85.7%, 57.1% and 42.9%, respectively.

## Discussion

BC is currently one of the tumors with the most rapid increase in incidence. At the time of diagnosis, approximately 15% of patients who present with bladder cancer will be found to have regional or distant metastasis [8–10]. Radical cystectomy with PLND and UD continues to be the gold standard for MIBC [11]. It is a clinical and therapeutic dilemma when these tumors are found to be unresectable (pN2-3 or pT4b) on surgical exploration. In this situation, chemotherapy and/or radiotherapy are the only treatment that has been shown to improve survival as described previously [12, 13]. However, systemic chemotherapy and radiotherapy may have severe side effects, such as significant hematologic, mucosal toxicity or gastrointestinal bleeding or perforation [14], or lead to treatment-related death [15].

Faysal A. Yafi et al. addressed the outcomes of thirty-one patients who underwent aborted RC due to unresectable disease. The outcome of patients with unresectable disease is dismal. The 2- and 5-year OS was 41% and 0%, respectively. They get the conclusion that patients who had an aborted cystectomy due to unresectable disease may benefit from PLND and upfront chemotherapy [11]. Recently, a study included 35 patients who had an aborted radical cystectomy for intraoperative findings of metastatic bladder cancer [16]. It showed a poor prognosis for these patients with a median time to cancer-specific survival of 26.5 months, and OS at one, two, and three years of 67%, 43%, and 18% respectively. Other several literatures reported that adjuvant radiotherapy also has the ability to improve the OS and PFS in patients with MIBC [14, 17]. From these results of previous studies, the survival enhancement was not very satisfying in combined chemoradiation or chemotherapy/radiotherapy alone.

Cryosurgery, also known as cryoablation, is widely used as primary or salvage treatment for various urological malignances like renal cell carcinoma (RCC) and prostate cancer (PC) in recent years [18–21]. It is reported that cryosurgery offers benefits with fewer complications, shorter hospitalization times, and allows for quicker convalescence in the treatment of RCC and PC [22–25]. Transperineal cryosurgery for prostate cancer has been carried out since 2006 in our hospital. Lian et al. addressed the preliminary results of 102 patients underwent primary cryoablation for clinically localized PCa from January 2006 to December 2009 in Nanjing Drum Tower Hospital. The early results of their study suggest that cryoablation offers a safe and effective alternative for the primary treatment of localized prostate cancer [24]. Recently, Zhou et al. analysed the results of a study of 23 patients with metastatic BC under percutaneous cryosurgery. No severe complications occurred in the study. The PFS of these patients was 14 ± 8 months, and indicated that percutaneous cryosurgery may be a safe and efficacious therapeutic option in the treatment of metastatic BC [5].

In the present study, we attempted to manage the aborted RC due to unresectable disease secondary to fixation to the pelvis and/or rectum (pT4b stage) and cryosurgery for these unresectable bladder cancer throuth transperineal approach. In this retrospective study, the longest 43 months of follow-up data from 7 patients obtained from our hospital’s database were analyzed to determine the safety and efficacy of this procedure in bladder cancer (Fig. 1). In the study, all cryosurgical procedures for bladder cancer were performed successfully, with no treatment-related deaths. No severe preoperative, intraoperative or postoperative complications were happened. The therapeutic effect of transperineal cryoablation was evaluated by means of PFS. The PFS of the 7 patients who underwent comprehensive cryosurgery for unresectable bladder cancer was 22.00 ± 14.61 months, which was better than that reported for cisplatin monotherapy and M-VAC combination chemotherapy [26]. The 1-, 2- and 3-year OS was 85.7%, 57.1% and 42.9% in the study, respectively. This study indicated that transperineal cryoablation may be an attractive alternative treatment for unresectable pT4b stage bladder cancer. This technique also could applicable to patients who could not tolerate cystectomy due to the minimal invasion.

According to our preliminary experience of transperineal cryosurgery for unresectable MIBC of 7 male patients, we believe that the following is the key to ensure cryotherapy successful. Firstly, the use of TRUS to monitor the appearance of the ice ball as well as to guide cryoprobe placement to avoid any injury to other tissues, especially rectum. Secondly, the placement of temperature monitoring probes around tumor and rectum to enhance the temperature information provided by TRUS. Moreover, the success of this procedure benefits from the previous experience of transperineal cryosurgery for prostate cancer in our hospital. Furthermore, patient selection is also very important.

In this study, we describe a cryosurgery alone, cryo-chemoradiation or cryo-chemotherapy combination as an alternative treatment in unresectable pT4b stage bladder cancer. We believe that reduction local tumor burden through cryosurgery and followed with chemoradiation or chemotherapy will benefit more than chemoradiation or chemotherapy alone for locally advanced BC. Moreover, we believe that patients with the pT4b stage unresectable disease will benefited more from this procedure than pN2-3 patients. However, the retrospective design raises the issue of potential selection bias which are likely to have influenced the results of the present study. Moreover, it is limited by its small series and single-institution study design. Future prospective, large-scale and long-term studies are needed in multiply centers.

## Conclusions

Transperineal cryoablation combination with chemoradiotherapy may be an option in the treatment of unresectable invasive bladder cancer due to unresectable disease secondary to fixation to the pelvis and/or rectum (pT4b) and these patients who could not tolerate cystectomy. The side effects were relatively mild and a PFS of 22.00 ± 14.61 months can be achieved. A larger number of cases is required, however, and long-term survival following this therapy and its role in combination with other therapies awaits further research.

## Abbreviations

BC: bladder cancer
CT: computed tomography
MIBC: muscle invasive bladder cancer
OS: overall survival
PFS: Progression free survival
PLND: pelvic lymph node dissection
RC: radical cystectomy
TRUS: transrectal ultrasound
TURBT: transurethral resection of bladder tumor
UD: urinary diversion
